# The Influence of Environmental Conditions on the Antagonistic Activity of Lactic Acid Bacteria Isolated from Fermented Meat Products

**DOI:** 10.3390/foods10102267

**Published:** 2021-09-25

**Authors:** Anna Łepecka, Piotr Szymański, Sylwia Rutkowska, Kinga Iwanowska, Danuta Kołożyn-Krajewska

**Affiliations:** 1 Department of Meat and Fat Technology, Prof. Waclaw Dabrowski Institute of Agriculture and Food Biotechnology—State Research Institute, Rakowiecka 36 St., 02-532 Warsaw, Poland; piotr.szymanski@ibprs.pl; 2 Department of Food Gastronomy and Food Hygiene, Institute of Human Nutrition Sciences, Warsaw University of Life Sciences—SGGW, Nowoursynowska 159c St., 02-776 Warsaw, Poland; sylwia.dobrzeniecka@op.pl (S.R.); kinga19940407@gmail.com (K.I.); danuta_kolozyn_krajewska@sggw.edu.pl (D.K.-K.)

**Keywords:** LAB strains, microorganism, antimicrobial, environmental stress, co-culture, meat

## Abstract

The aim of this study was to determine the impact of environmental conditions on the antimicrobial properties of 21 lactic acid bacteria strains in the selected indicator bacteria. To assess the antimicrobial activity of the whole bacteria culture (WBC), the agar well diffusion method was used. The interference of LAB strains with the growth of the selected indicator bacteria was evaluated by incubating co-cultures in the food matrix. Based on the conducted research, it was found that environmental conditions have a significant impact on the antimicrobial activity of lactic acid bacteria strains. The highest antimicrobial activity was recorded under optimal conditions for the development of LAB, the incubation time being different depending on the indicator strain used. The tested LAB strains were characterized by a high ability to inhibit indicator strains, especially in the food matrix. These results led us to further characterize and purify the antimicrobial compound produced by lactic acid bacteria taking into account changing environmental conditions.

## 1. Introduction

Lactic acid bacteria (LAB, LABs) exhibit antimicrobial properties against Gram(+) and Gram(-) bacteria. There are several mechanisms of antimicrobial action, and not all of them have been fully explained. These mechanisms can be competition for the adherence site, competition for nutrients, and/or production of non-specific antibacterial substances. LAB may cause the inactivation of other microorganisms, mainly through the production of lactic acid, acetic acid, propionic acid, diacetyl, acetaldehyde, H_2_O_2_, bacteriocins, exopolysaccharides, fatty acids, and amino acids metabolites [1,2]. Typically, bacteria produce more than one antimicrobial substance, which increases the spectrum of their activity [3]. LAB strains can function as microbial barriers against gastrointestinal pathogens through the competitive exclusion of pathogen binding, modulation of the host’s immune system, and production of inhibitory compounds [4]. In recent years there has been a search for new antimicrobial substances that are safe, natural and eco-friendly. LAB are very potential candidates because of their widely known positive healthy effects [5,6,7,8].

By securing favorable environmental conditions, LAB inhibit the development of pathogenic microflora. In order to understand the most favorable conditions for the production of antimicrobial substances, and to examine the impact of stress on their synthesis, a detailed analysis should be carried out, taking into account many factors. According to other studies [9,10], cell culture conditions deviating from the optimum increased the amount of produced antimicrobial compounds. Environmental stress can lead to the incorporation of defense mechanisms into LAB cells, and thereby intensify the production of protein substances which are antagonistic to other bacteria, in order to ensure the survival of cells under unfavorable conditions. Probably, a modification of environmental conditions may increase the effectiveness of the search for strains with strong antimicrobial properties. In fact, the antagonistic properties of individual microorganisms are difficult to assess. According to Mataragas et al. [11], the production of antimicrobial substances was strongly affected by the manipulation of temperature and pH. In order to effectively use antimicrobial compounds, it is necessary to optimize production conditions by controlling technological parameters and the environment. A detailed analysis of the biochemical and technological properties of these types of compounds can thus explain their low activity.

The aim of this study was to determine the impact of environmental conditions (incubation time, temperature, and pH) on the antimicrobial properties of LAB strains isolated from fermented meat products on the selected indicator bacteria and study the effects of LAB and pathogens against each other in experimental co-culture.

## 2. Materials and Methods

### 2.1. Strain Collection and Bacterial Growth Conditions

A total of 21 LAB strains were previously isolated from organic fermented meat products [12]. The list of LAB strains is presented below in Table 1.

The isolates were identified by the sequencing of 16S rRNA (Genbank accession were noted) and were tested for probiotic properties (survival studies, safety assessment, enzyme and antimicrobial activities). The strains come from a collection of the Department of Food Gastronomy and Food Hygiene WULS-SGGW. The foodborne commensal strains and pathogens were selected as indicator strains based on previous studies [14,15,16,17]. *Escherichia coli* ATCC 10536, *Salmonella enteritidis* ATCC 13076, *Listeria monocytogenes* ATCC 19111, *Staphylococcus aureus* ATCC 25923 and *Bacillus cereus* ATCC 14579 were used as indicators and were received from American Type Culture Collection (ATCC).

LAB strains were stored in MRS broth (de Man, Rogosa and Sharpe broth, LabM Ltd., Bury, UK) and the indicator strains were stored in nutrient broth (Nutrient broth No. 2, LabM Ltd., Bury, UK) at −80 °C with 20% glycerol (w/v, Merck KGaA, Darmstadt, Germany). LAB isolates and indicator bacteria were cultivated in MRS and nutrient broth, respectively, at 37 °C for 24 h under anaerobic conditions before each experiment. All experiments were performed in triplicate.

### 2.2. Antagonistic Activity

To assess the antimicrobial activity of whole bacteria culture (WBC), the agar well diffusion method was used according to Soleimani et al. and Ołdak et al. [15,18]. A 0.5 McFarland standard suspension of LAB strain in MRS broth with a turbidity of 1.5 × 10^8^ CFU mL^−1^ was used to standardize the inoculum. Sterile Petri dishes with Mueller-Hinton agar I (MHA, LabM Ltd., Bury, UK) were inoculated with 200 µL of a 24 h culture of the indicator bacteria in nutrient broth (concentration ≈ 10^6^ CFU mL^−1^). Wells with a diameter of 5.5 mm were then cut out and filled with 50 µL of WBC. The plates were incubated at 37 °C for 24 h and the inhibition zone diameter (X) was determined as follows:

X = D − d(1)

where X—inhibition zone diameter (mm); D—total growth inhibition zone diameter (mm); d—the well diameter (5.5 mm).

### 2.3. Study of the Effect of Incubation Time on Antagonistic Activity

The effect of incubation time on the microbiological activity of LAB strains was tested at four time intervals (24, 48, 72, and 96 h) at a temperature of 37 °C [10]. The cultures were performed as described above in Section 2.2.

### 2.4. Study of the Effect of Temperature on Antagonistic Activity

The effect of temperature on the microbiological activity of LAB strains was tested at three temperatures. The strains were incubated in MRS broth under modified temperatures: 20, 30 and 37 °C for 24 h and then used for testing [9]. In addition, an experiment was carried out using strains subjected to stress conditions. The strains were first incubated at 37 °C for 24 h and then placed in a water bath at 55 or 100 °C during 10 min [9]. These temperatures are often considered critical for microbial growth and were selected on the basis of the cited publication. The cultures were performed as described above in Section 2.2.

### 2.5. Study of the Effect of pH on Antagonistic Activity

Strains were incubated for 24 h at 37 °C in nutrient broth with a different pH of 4.0, 5.7, 6.4, and 8.0 [9]. To obtain a pH of 4.0 and 5.7, concentrated 1M HCl (Merck KGaA, Darmstadt, Germany) was gradually added to the MRS broth. The pH of 6.4 was obtained by preparing the MRS broth according to the instructions on the preparation label. The pH of 8.0 was obtained by pipetting 1M NaOH (Merck KGaA, Darmstadt, Germany). The pH changes were measured using the Elmetron CP-511 pH-meter (Elmetron Sp.j., Zabrze, Poland). Then, the modified MRS broths were sterilized for 15 min at 121 °C. LAB strains were incubated in modified MRS broths for 24 h at 37 °C and then used for testing. The cultures were performed as described above in Section 2.2.

### 2.6. Co-Culture Assay

The interference of LAB strains with the growth of the selected indicator bacteria was evaluated by incubating a co-culture of both strains and comparing the recovered cells with those obtained from pure cultures of both strains. Based on the results of statistical analysis, two strains were selected for co-culture studies. The UHT milk was selected as the food matrix because of the pH (6.6) similar to the pH of the MRS broth (6.4; culture broth for LAB). Three indicator strains were selected, whose growth was mostly inhibited by the tested LAB (selection based on statistical analysis). The temperature of 37 °C was the optimal condition for the growth of most of the foodborne pathogens. The same time intervals indicated in the previous section were used.

Co-culture tests were performed according to Ołdak et al. [15] with slight modifications. In total, 50 mL of UHT milk was inoculated with the LAB strain to induce fermentation. A 0.5 McFarland standard suspension of LAB strain in MRS broth with a turbidity of 1.5 × 10^8^ CFU mL^−1^ was used to standardize the inoculum. The milk was incubated for 24 h at 37 °C. Then, after 24 h, 1 mL of indicator bacteria in nutrient broth (concentration 10^6^ CFU mL^−1^) was added to the fermented milk and placed at a temperature of 37 °C (RS—research sample). The following test designations were used: RS-LAB—research sample, *Lb. plantarum* SCH4 or BAL7; RS-Lm—research sample, *L. monocytogenes* ATCC 19111; RS-Ec—research sample, *E. coli* ATCC 10536; RS-Sa—research sample, *S. aureus* ATCC 25923. The samples inoculated only with the indicator strains or only with the *Lb. plantarum* strains were also prepared as controls (CS—control sample). The following test designations were used: CS-LAB—control sample, *Lb. plantarum* SCH4 or BAL7; CS-Lm—control sample, *L. monocytogenes* ATCC 19111; CS-Ec—control sample, *E. coli* ATCC 10536; CS-Sa—control sample, *S. aureus* ATCC 25923. In the case of the incubation of the milk with the indicator bacteria, no fermentation of milk was carried out. Microbiological cultures were performed at time 0 and after 24, 48, 72, and 96 h of the incubation.

LAB were plated on MRS agar (de Man, Rogosa and Sharpe agar, LabM Ltd., Bury, UK), *L. monocytogenes* was plated on PALCAM agar (LabM Ltd., Bury, UK), and *E. coli* was plated on TBX agar (Tryptone Bile X-Glucuronide Agar, LabM Ltd., Bury, UK), *S. aureus* was plated on Baird-Parker agar with Egg Yolk Tellurite Supplement (LabM Ltd., Bury, UK). The plates with MRS agar, Baird-Parker agar, and PALCAM agar were incubated at 37 °C for 24–48 h and the plates with TBX agar were incubated at 44 °C for 24 h. After incubation, colonies on each solid medium were counted and the results obtained were transformed into a logarithmic scale. The number of LAB and the number of indicator bacteria were checked in parallel. The results were presented on linear charts.

### 2.7. Statistical Analysis

The test results were given after averaging the results from three replicates of the zones of the inhibition areas. The standard deviation was calculated. A one-way analysis of variance (ANOVA) and post-hoc analyses (Tukey’s HSD) were performed at the significance level of *p* < 0.05. The statistical results were analyzed in STATISTICA 13 (TIBCO Software Inc., Palo Alto, California, USA) program.

## 3. Results and Discussion

Antimicrobial activity may be affected by several factors, including the pH value of the environment, temperature, cell growth phase, co-culture incubation, culture medium composition, aerobic conditions, presence of surface tension reducing agents, and the addition of mineral salts [9,19,20,21].

**Figure 1:**
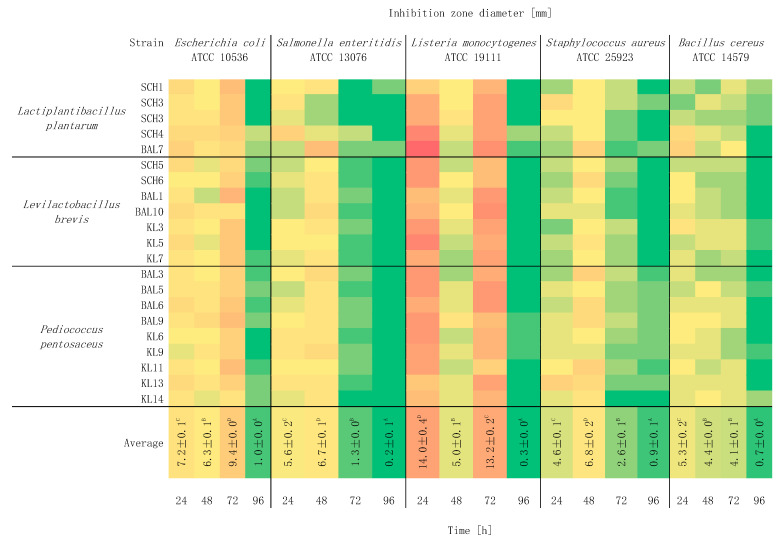
Heatmap about the influence of incubation time on the antimicrobial activity of LAB strains and against indicator bacteria (*n* = 3). Means in the same column followed by different letters (A–D) represent significant differences (*p* < 0.05) for each incubation time and indicator bacteria.

Figure 1 presents the impact of incubation time on the antimicrobial activity of LAB strains. Based on the obtained results, it was found that the incubation time had a significant effect on the antimicrobial properties of the strains (*p* < 0.05), but the use of an indicator microorganism was also important. The highest inhibition of *E. coli* (*p* < 0.05) was observed after 72 h. In the case of *S. enteritidis*, significantly different growth inhibition was observed after 24 and 48 h. The greatest inhibition of *L. monocytogenes* growth was observed after 24 and 72 h (*p* < 0.05). With respect to *S. aureus*, a significant inhibition of bacterial growth was observed after 48 h, and inhibition of *B. cereus* after 24 h. Extending the incubation time to 96 h resulted in a significant decrease in antagonistic activity.

Bacteria are affected by the surrounding environment and they react dynamically to changes in external conditions. The number of cells in the culture medium determines the antagonistic activity of the lactic bacteria. The greatest synthesis of microbial agents occurs in the terminal phase of logarithmic growth and at the beginning of the stationary phase [22]. The logarithmic growth phase is characterized by the most intensive cell growth at a constant rate. The duration of this phase is determined by several factors, such as the amount of nutrients, the presence of toxic substances, cell metabolism products, pH value, temperature, oxygen content and type of culture. On the other hand, the stationary phase is characterized by a fixed number of cells, which is the result of the balance between the number of live and dead cells. Not always is the amount of biomass produced coupled with the production of microbial agents. Depending on the incubation temperature of the strain, the synthesis may occur earlier or later [10]. It has been reported that stress responses vary depending on the logarithmic growth phase of LAB, i.e., cells in the stationary phase develop more general resistance to various types of stresses compared to cells in the exponential growth phase [23,24]. The effect of different cultivation conditions, in particular incubation time, carbon, nitrogen and mineral sources, on EPS (Exopolysaccharides) production often varies from strain to strain [25].

**Figure 2:**
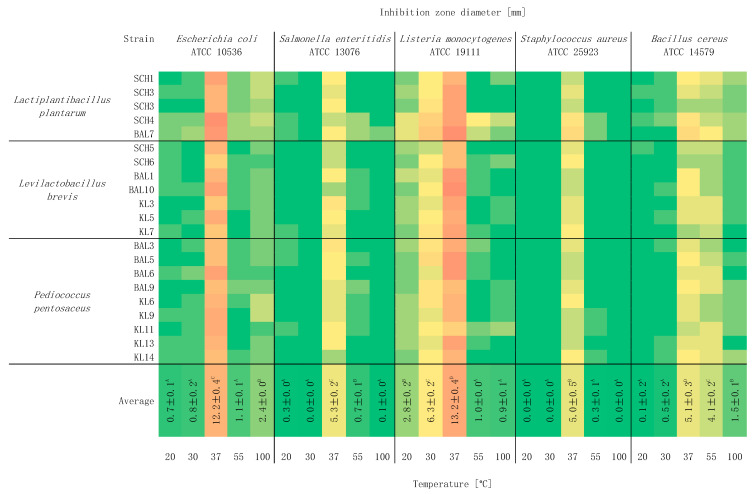
Heatmap about the influence of temperature on the antimicrobial activity of LAB strains and against indicator bacteria (*n* = 3). Means in the same column followed by different letters (A–D) represent significant differences (*p* < 0.05) for each temperature and indicator bacteria.

Figure 2 shows the effect of temperature on the antimicrobial activity of LAB strains. This research showed that the statistically significant inhibition areas of the indicator bacteria growth were shown at 37 °C (*p* < 0.05). At 20 and 30 °C temperatures, a slight inhibition of the indicator bacteria growth was observed. Interestingly, LAB strains subjected to stress conditions (55 and 100 °C) still showed quite strong antimicrobial activity. The strongest antimicrobial properties at 55 and 100 °C were found against *B. cereus* ATCC 14579 and *E. coli* ATCC 10536, respectively. The lowest, not statistically different, antagonistic properties were found at 20 °C and subjected to short-term exposure at 100 °C. The question, therefore, arises whether dead cell metabolites also have antagonistic properties [26,27,28]. Understanding the response to adverse temperature conditions is a task that requires the consideration of many factors that could modulate bacterial behavior. Therefore, studies about the effects of different incubation temperatures on cell survival should be extended to studies on adaptability to moderate stresses, which may increase resistance to more adverse conditions, and molecular studies of changes in the profile of produced proteins are needed. High temperatures may induce the production of various types of proteins, such as heat shock proteins (HSPs) [29].

The growth temperature for mesophilic LAB is 15–45 °C [30]. A temperature of 37 °C is optimal for the development of most human pathogenic bacteria. The optimal temperature for LAB development does not always coincide with the temperature where the synthesis of microbial agents is the most intensive. Firstly, lower temperatures favor lower LAB biomass production, which may favor the production of non-specific metabolites [31]. On the other hand, at a higher temperature, more energy can be retained in the cell, which in turn facilitates the synthesis of antimicrobial agents [19]. High temperatures can also have a beneficial effect on metabolite production. Bacteria exposed to stress increase the production of protective factors to preserve vitality and metabolic functions, thereby determining the production of microbial agents. In many cases, bacteriocins are the most intensively produced by LAB at the optimum temperature for their growth [22,27,28,29,30,31,32,33,34,35,36]. However, this study also presents cases when the environmental temperature exceeds the optimum triggered the defense mechanisms of cells of other LAB species, which was associated with increased production of compounds with antagonistic properties.

**Figure 3:**
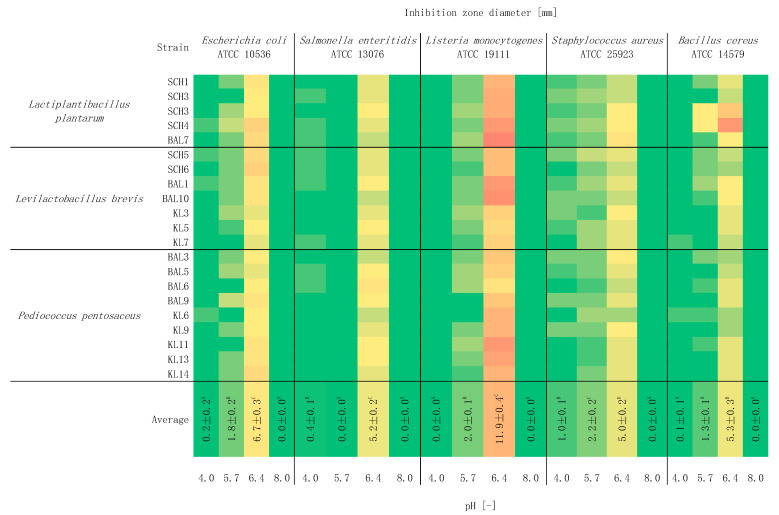
Heatmap about the influence of pH on the antimicrobial activity of LAB strains and against indicator bacteria (*n* = 3). Means in the same column followed by different letters (A–D) represent significant differences (*p* < 0.05) for each temperature and indicator bacteria.

The impact of pH on the antimicrobial activity of LAB strains is shown in Figure 3. The tested strains have the strongest antimicrobial properties at pH of 6.4 against all of the tested microorganisms with the strongest antimicrobial activity against *L. monocytogenes* ATCC 19111 (*p* < 0.05). A change in the pH of the environment below 6.4 resulted in a decrease or total disappearance of the antimicrobial properties of strains. Lowering the pH to 5.7 value significantly reduced the created zones of inhibition. On the other hand, a further decrease in the pH of the environment to 4.0 and an increase outside the optimum to 8.0 resulted in a decrease in antagonist activity at the same level. A decrease in the antimicrobial activity of CFS of *Lb. casei*, *Lb. acidophilus*, and *Lb. salivarius* strains at a pH above 7 was observed by Moradi et al. [37]. A high percentage of residual antimicrobial activity against *L. monocytogenes* was observed at a pH of 4.0 and 5.0.

LAB strains have optimum pH environmental conditions in the range of 5.4–6.4. However, it has been found that the isolates of this species have survived even at a pH of 2.8 or 10.0 [38]. Thanks to their ability to regulate intracellular pH, they can survive in a relatively low pH environment (low concentration of hydrogen ions damages the natural mechanism of pH gradient). As the enzymatic reactions are controlled by pH value, the decrease of pH may have positive effects on the synthesis of antimicrobial compounds, such as bacteriocins, through increasing the pools of essential metabolites, including ATP. However, enzymatic reactions are regulated by pH and temperature, which in consequence can lead to their inhibition and accordingly to the inhibition of the synthesis of antimicrobial compounds [38].

**Figure 4:**
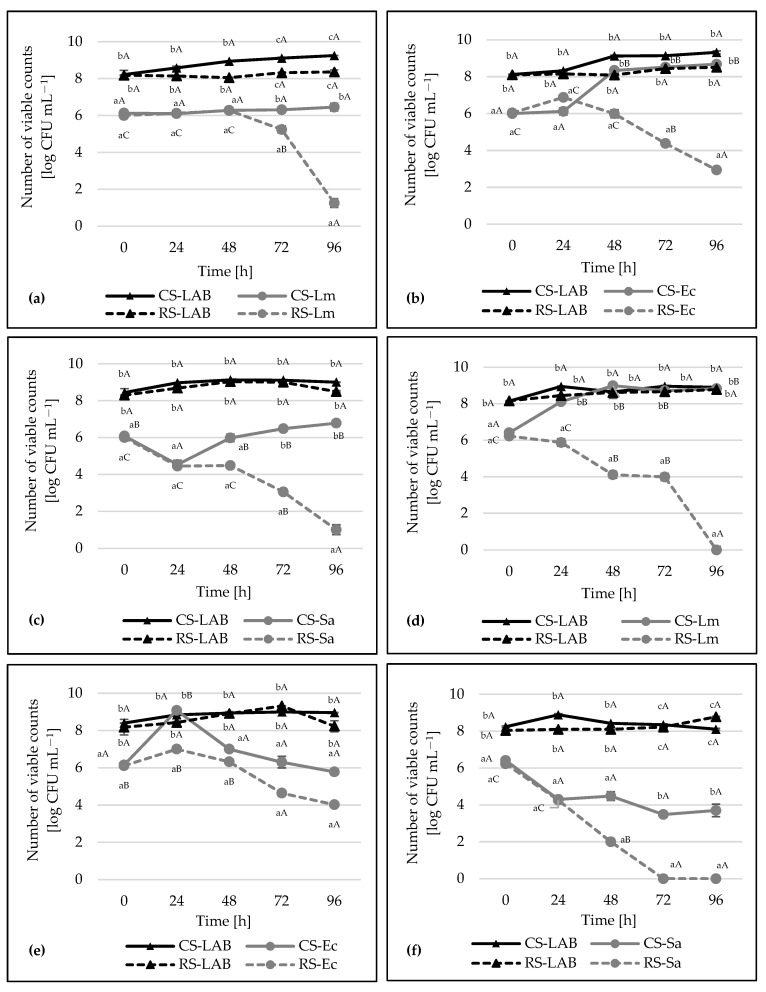
(**a**) Impact of *Lb. plantarum* SCH4 on the viability of *L. monocytogenes* ATCC 19111 in milk; (**b**) impact of *Lb. plantarum* SCH4 on the viability *E. coli* ATCC 10536 in milk; (**c**) impact of *Lb. plantarum* SCH4 on the viability of *S. aureus* ATCC 25923 in milk; (**d**) impact of *Lb. plantarum* BAL7 on the viability of *L. monocytogenes* ATCC 19111 in milk; (**e**) impact of *Lb. plantarum* BAL7 on the viability of *E. coli* ATCC 10536 in milk; (**f**) impact of *Lb. plantarum* BAL7 on the viability of *S. aureus* ATCC 25923 in milk; explanatory: CS-LAB—control sample, *Lb. plantarum* SCH4 or BAL7; RS-LAB—research sample, *Lb. plantarum* SCH4 or BAL7; CS-Lm—control sample, *L. monocytogenes* ATCC 19111; RS-Lm—research sample, *L. monocytogenes* ATCC 19111; CS-Ec—control sample, *E. coli* ATCC 10536; RS-Ec—research sample, *E. coli* ATCC 10536; CS-Sa—control sample, *S. aureus* ATCC 25923; RS-Sa—research sample, *S. aureus* ATCC 25923; the graph shows the log bacterial cell count (CFU mL^−1^) with standard deviation. Means in the same row followed by different uppercase letters within the same sample in different times are significantly different (*p* < 0.05); means in the same column followed by different lowercase letters within samples in the same time are significantly different (*p* < 0.05).

Several studies [39,40,41,42] have been carried out on the application of the pure culture of LAB to control the growth of foodborne microorganisms. According to the statistical analysis, *Lb. plantarum* SCH4 and BAL7, *L. monocytogenes* ATCC 19111, *E. coli* ATCC 10536, and *S. aureus* ATCC 25923 were selected for a further study. The relationship between strains and environmental conditions are presented in Figure 4a–f.

Co-culture is a system containing two distinct types of cells and allowing the growth of both types of microorganisms [43]. Tabasco et al. [44] reported that antimicrobial compound synthesis and enhanced antimicrobial properties may occur, provided that bacteria are used in co-culture with starter microorganisms (e.g., starter yogurt culture *Str. thermophilus* and *Lb. delbrueckii* subsp. *bulgaricus*), or in co-culture with other Gram-positive and Gram-negative bacteria strains or the presence of the supernatant from their culture. The competition can have a positive impact on the production of protein substances.

Figure 4a–c shows the effect of *Lb. plantarum* SCH4 on the viability of indicator strains. As a result of this study, a statistically significant inhibitory effect on *L. monocytogenes* ATCC 19111, *E. coli* ATCC 10536, and *S. aureus* ATCC 25923 was observed after 72 h of incubation. Compared to control sample (CS-Lm), a decrease in the number of *L. monocytogenes* ATCC 19111 was observed after 72 h (1 log CFU mL^−1^) and after 96 h (5 log CFU mL^−1^) of incubation. In the case of *E. coli* ATCC 10536 a non-statistically significant increase in the number of indicator strains was observed after 24 h and a decrease in number after 48 h. After 72 h, a statistically significant (*p* < 0.05) decrease in the number of *E. coli* ATCC 10536 was noted (about 2 log CFU mL^−1^) compared to the initial number (about 6 log CFU mL^−1^). After 96 h, the number of *E. coli* ATCC 10536 cells decreased to 3.00 log CFU mL^−1^. In control sample (CS-Ec), a significant increase was observed (8 log CFU mL^−1^). The number of *S. aureus* ATCC 25923 significantly (*p* < 0.05) decreased after 72 h (3.00 log CFU mL^−1^), and after 96 h (1.00 log CFU mL^−1^). The number of bacteria in control sample (CS-Sa) increased steadily and after 96 h was almost 7 log CFU mL^−1^. The number of viable cells of *Lb. plantarum* SCH4 in control sample (CS-LAB) and research sample (RS-LAB) throughout the incubation was 8–9 log CFU mL^−1^. In summary, the significant effect of *Lb. plantarum* SCH4 on inhibiting the indicator strain growth in the food matrix was demonstrated.

The *Lb. plantarum* BAL7 strain showed even more strong antimicrobial activity (Figure 4d–f). A statistically significant (*p* < 0.05) decrease in the number of *L. monocytogenes* ATCC 19111 was observed after 48 and 72 h (to 4.00 log CFU mL^−1^), and the complete inhibition of growth was observed after 96 h. The number of viable cells of *L. monocytogenes* ATCC 19111 in the control sample (CS-Lm) after 48, 72, and 96 h was about 9 log CFU mL^−1^. In the case of *E. coli* ATCC 10536, a significant decrease in bacterial numbers was observed after 72 and 96 h (≈5 and 4 log CFU mL^−1^, respectively). The number of *E. coli* ATCC 10536 in the control sample (CS-Ec) also decreased, but to a lesser extent than in the test sample (RS-Ec). In contrast, the largest inhibitory effect of *Lb. plantarum* BAL7 strain was in relation to *S. aureus* ATCC 25923. A statistically significant (*p* < 0.05) decrease in the number of bacterial cells was observed after 48 h. After 72 h, the growth of *S. aureus* ATCC 25923 cells was completely inhibited. In the control (CS-Sa), a reduction in the number of *S. aureus* ATCC 25923 was observed after 24 h, followed by a bacterial cell count of ≈4 log CFU mL^−1^ for the remaining incubation time. The *Lb. plantarum* BAL7 strain was characterized by the high ability to inhibit the indicator bacteria growth in the food matrix.

The capability of the *Lactobacillus* isolates to inhibit the growth of *S. typhi* was evaluated in a co-culture experiment by Abdel-Daim et al. [4]. The results showed that nine *Lactobacillus* strains inhibited the growth of *Salmonella* after 24 h of incubation. However, the growth of tested *Lactobacillus* isolates was not affected by the simultaneous presence of *Salmonella* typhi. Similar test results were obtained by Ołdak et al. [15], where *Lb. plantarum* strains isolated from regional cheeses, showed the greatest antimicrobial activity after 24 and 64 h of incubation in co-cultures. Based on our co-culture results, a statistically significant inhibition of indicator strain growth was achieved after 48 or 72 h, depending on the type of the strain tested. However, it should be noted that the high-dose inoculum of indicator strains was used (~10^6^ CFU mL^−1^). In the study of Shah et al. [43] it was observed that in the co-culture, LAB strains showed a significant reduction of growth of *E. coli* after 24 h of incubation. Similar to our findings, Kang et al. [45] had also observed that *Lb. salivarius* and *Lb. fermentum* strains led to a significant reduction in *S. aureus*, but after 24 h. Koohestani et al. [46] showed that the application of CFS of *Lactobacillus* strains as antibacterial and biofilm removal compounds could be very suitable to control the growth of foodborne pathogens. Antimicrobial properties, production of bacteriocins or other antimicrobial substances can have potential preserving natural food. In order to increase the productivity of these substances, it is necessary to know the optimal conditions for their production. The production of antimicrobial substances, including bacteriocins, is strictly dependent on conditions such as environmental pH, temperature and incubation time. These conditions may differ from precisely established ones and be optimal for a given species of bacteria [47]. These factors could affect the viability and functional properties of microorganisms. Defense mechanisms against stress conditions were developed by LAB in order to survive when exposed to sudden environmental changes [48].

## 4. Conclusions

According to Parlindungan et al. [49] due to the growing interest in probiotics, it becomes crucial to characterize new probiotic strains with unique probiotic properties. LAB strains isolated from fermented meat products showed a strong activity towards indicator bacteria. The experiments in this study were conducted both on the microbiological medium and the food matrix. Based on our results, it was found that the largest inhibitory effect of *Lb. plantarum* strains on indicator bacteria were observed after 24 and 72 h of the incubation, at 37 °C and with a pH of 6.4. The greatest synthesis of microbial agents occurs in the terminal phase of logarithmic growth and at the beginning of the stationary phase. The temperature of 37 °C and pH of 6.4 are considered optimal for the growth of LAB and it was under these conditions that the greatest antimicrobial properties were found. Antimicrobial properties have also been observed in suboptimal conditions, indicating the potential for the production of antimicrobial substances under stress conditions.

This study will contribute to a better understanding of the influence of environmental factors on the behavior of the LAB strains. The tested isolates with potential probiotic properties showed promising antagonistic activity against some indicator strains. As antimicrobial activity is strain-dependent, more thorough research into compounds with antimicrobial properties produced by the studied strains is needed.

The results of this study indicate that LAB are capable of exhibiting antimicrobial activity under various environmental conditions, both optimal and non-optimal, which indicates the potential for the production of some antimicrobial substances. A modification of environmental conditions may increase the effectiveness of the search for strains with strong antimicrobial properties.

## Figures and Tables

**Table 1:** The tested LAB strains and new nomenclature of LAB.

Tested LAB Strains [12]	GenBank Accession [12]	New Nomenclature of LAB [13]
*Lactobacillus plantarum* SCH1	KX 014848	*Lactiplantibacillus plantarum* SCH1
*Lactobacillus plantarum* SCH2	KX 014847	*Lactiplantibacillus plantarum* SCH2
*Lactobacillus plantarum* SCH3	KX 021365	*Lactiplantibacillus plantarum* SCH3
*Lactobacillus plantarum* SCH4	KX 021366	*Lactiplantibacillus plantarum* SCH4
*Lactobacillus plantarum* BAL7	KX 021348	*Lactiplantibacillus plantarum* BAL7
*Lactobacillus brevis* SCH5	KX 021367	*Levilactobacillus brevis* SCH5
*Lactobacillus brevis* SCH6	KX 021368	*Levilactobacillus brevis* SCH6
*Lactobacillus brevis* BAL1	KX 021369	*Levilactobacillus brevis* BAL1
*Lactobacillus brevis* BAL10	KX 021350	*Levilactobacillus brevis* BAL10
*Lactobacillus brevis* KL3	KX 021351	*Levilactobacillus brevis* KL3
*Lactobacillus brevis* KL5	KX 021352	*Levilactobacillus brevis* KL5
*Lactobacillus brevis* KL7	KX 021354	*Levilactobacillus brevis* KL7
*Pediococcus pentosaceus* BAL3	KX 021370	Unchanged name
*Pediococcus pentosaceus* BAL5	KX 021346	Unchanged name
*Pediococcus pentosaceus* BAL6	KX 021347	Unchanged name
*Pediococcus pentosaceus* BAL9	KX 021349	Unchanged name
*Pediococcus pentosaceus* KL6	KX 021353	Unchanged name
*Pediococcus pentosaceus* KL9	KX 021361	Unchanged name
*Pediococcus pentosaceus* KL11	KX 021362	Unchanged name
*Pediococcus pentosaceus* KL13	KX 021363	Unchanged name
*Pediococcus pentosaceus* KL14	KX 021364	Unchanged name

## Data Availability

Not applicable.
